# Enhanced bioenergy and nutrients recovery from wastewater using hybrid anodes in microbial nutrient recovery system

**DOI:** 10.1186/s13068-022-02116-y

**Published:** 2022-02-17

**Authors:** Kanwal Shahid, Deepika Lakshmi Ramasamy, Parminder Kaur, Mika Sillanpää, Arto Pihlajamäki

**Affiliations:** 1 Department of Separation Science, School of Engineering Science, Lappeenranta-Lahti University of Technology, Sammonkatu 12, 50130 Mikkeli, Finland; 2 Department 4MAT, Université Libre de Bruxelles, Avenue F.D. Roosevelt, 50. CP 165/63, 1050 Bruxelles, Belgium; 3 Department of Chemical and Metallurgical Engineering, Aalto University, Aalto, Finland; 4 Chemistry Department, College of Science, King Saud University, Riyadh, 11451 Saudi Arabia; 5 School of Resources and Environment, University of Electronic Science and Technology of China (UESTC), NO. 2006, Xiyuan Ave., West High-Tech Zone, Chengdu, Sichuan 611731 People’s Republic of China; 6 Faculty of Science and Technology, School of Applied Physics, University Kebangsaan Malaysia, 43600 Bangi, Selangor Malaysia; 7 School of Chemistry, Shoolini University, Solan, Himachal Pradesh 173229 India; 8 Department of Biological and Chemical Engineering, Aarhus University, Nørrebrogade 44, 8000 Aarhus C, Denmark

**Keywords:** Hybrid anodes, Air cathode, Microbial fuel cell, Microbial nutrient recovery cell, Nutrients recovery

## Abstract

**Background:**

The combined microbial fuel cell–microbial nutrient recovery system has lately been thoroughly explored from an engineering standpoint. The relevance of microbial communities in this process, on the other hand, has been widely underestimated.

**Results:**

A lab-scale microbial nutrients recovery system was created in this work, and the microbial community structure was further defined, to give a thorough insight into the important microbial groups in the present system. We reported for the first-time different hybrid anodes of activated carbon and chitosan that were used in the microbial nutrient recovery system for bioenergy production, and, for the removal of COD and recovery of nutrients present in the wastewater. The hybrid anodic materials were studied to adapt electrochemically active bacteria for the recovery of nutrients and energy generation from wastewater without the need for an external source of electricity. The potential of the created hybrid anodes in terms of nutrients recovery, chemical oxygen demand elimination, and energy generation from municipal wastewater was thoroughly examined and compared with each other under similar operating conditions. When the COD loading was 718 mg/L, a total COD removal of ~ 79.2% was achieved with a hybrid activated carbon and chitosan anode having an equal ratio after 10 days of the operation cycle. The maximum power density estimated for hybrid anode (~ 870 mWm^−2^) was found.

**Conclusion:**

Overall, this work reveals a schematic self-driven way for the collection and enrichment of nutrients (~ 72.9% phosphorus recovery and ~ 73% ammonium recovery) from municipal wastewater, as well as consistent voltage production throughout the operation.

**Supplementary Information:**

The online version contains supplementary material available at 10.1186/s13068-022-02116-y.

## Background

Excessive agricultural production has resulted in higher quantities of nutrients (e.g., phosphate and ammonium) in aquatic ecosystems, as well as uncontrolled wastewater discharges, in recent years [1]. This could lead to the eutrophication of aquatic systems, which has negative consequences for human health and the environment [2]. Nutrient discharge limits are becoming more rigorous, which may make their removal in the wastewater treatment process more difficult. The active sludge process, chemical precipitation, nitrification–denitrification, and other traditional nutrient removal procedures are only a few examples used previously for this purpose [3]. However, nutrient removal may not be possible in a low-carbon, low-energy, and resource-recycling wastewater management system [4]. All living creatures require ammonium and phosphate because they are fundamental components in biological production (e.g., proteins).

Recent research has revealed a significant demand for ammonium and phosphate, which are utilized in fertilizer manufacturing, and this condition is attributable to industrial nutrient supply limitations, which is particularly concerning taking into consideration the world's growing population [5]. Large levels of nutrients have been discovered in wastewater sources, including not just wastewater, but also wastewater sludge, which is now considered a valuable source of nutrients. As a result, nutrient recovery from wastewater might make wastewater treatment more sustainable, lower the costs of nutrient removal (e.g., less surplus sludge creation), and supply more fertilizers for food production [6]. Many technologies, including classic methods like chemical precipitation and adsorption, as well as more sophisticated approaches like bioelectrochemical systems and osmotic membrane bioreactors (OMBRs), have been researched for their efficacy in nutrient recovery [7]. Apart from that, nutrients may be recovered from the wastewater during treatment in the liquid phase, for example, anaerobic digestion supernatant, and sludge dewatering filtrate and in the sludge phase, i.e., dry excess sludge and sewage sludge ash [8]. Most nutrient recovery technologies are utilized in the liquid phase, although wet-chemical and thermochemical treatments can extract phosphate from the sludge phase and subsequently interact with other recovery processes. Furthermore, using a biological technique, phosphate may be transported from the liquid phase to the sludge phase for recovery [9]. Many studies published in the recent decade offered thorough information on nutrient recovery from wastewater in terms of processes, the impact of many significant elements, future directions, and so on, but only a few focused on the economic aspects. Researchers are focusing on the development of waste-to-energy recovery systems to use wastewater as a possible renewable energy resource. The MFC is a waste-to-energy recovery system that uses bacteria as a biocatalyst to transform energy held in organics from the waste substrate into electrical output through different electrochemical reactions [10].

MFC technology is generally recognized as an innovative bioelectrochemical process that employs respiring microorganisms to extract energy from wastewater having high levels of organic matter [11]. Furthermore, MFC has been shown to improve wastewater substrate breakdown to extract renewable energy (with a net positive output) that was previously impossible to handle in an anaerobic environment [12]. As a result, MFC technology development is critical because it can give a cost-effective alternative to present and expensive water treatment methods [13]. An anode and a cathode are the essential components of MFC. Ion-exchange membranes (IEMs) are used to divide a cell into compartments [14]. As a result, the cell may be built in a variety of ways (single-chamber, double-chamber, and more) depending on the application [15]. Because of their good qualities, such as wide surface area, increased electrical conductivity, and mechanical strength, activated carbon (AC) and for biodegradability and biocompatibility of chitosan beads were chosen as the anode material for the establishment of microbial biofilm [16] in previous work by the researcher [17–19]. The discovery of novel anode materials that are biocompatible, cost-effective, and can speed efficient electron transport from the biofilm-assisted anode to the cathode is continually being explored [20].

Several studies have shown that the MFC system may be used as a microbial electrolysis cell (hydrogen generation), a microbial desalination cell, and a microbial electrosynthesis cell (CO_2_ reduction) in recent years [21]. Numerous research has lately been conducted to convert the MFC system to an MNRC system. [22]. Previous studies have mostly concentrated on using AC and chitosan beads separately in MFC for energy production and bacterial culture flourishing and further in the MNRC system for the recovery of nutrients and COD reduction [17]. However, the research works based on MNRC technology are limited, and additional experimental supports are needed to have a better understanding of energy conversion, organic matter degradation, and the ion/water transport process. Furthermore, easy setup and the use of low-cost components are two key criteria in the effective deployment of MNRC technology in the real world [23].

In this study, to realize resource utilization of wastewater, hybrid anodes of AC and chitosan were used along with wastewater for the culture of bacteria in MNRC technology to perform the treatment of wastewater, recovery of bioenergy present in the wastewater and COD, and nutrients removal simultaneously. Different hybrids of chitosan and activated carbon have been prepared to explore the growth of microorganisms and power generation in the MFC. The removal performance of COD, phosphate, ammonium, and nitrate from the wastewater using different hybrid anode in the MNRC process were studied. Microbial community analysis was performed to characterize the development of anodic microbes and power generation during wastewater treatment.

## Results and discussion

### Characterization of the hybrid’s anodic materials

**Fig. 1:**
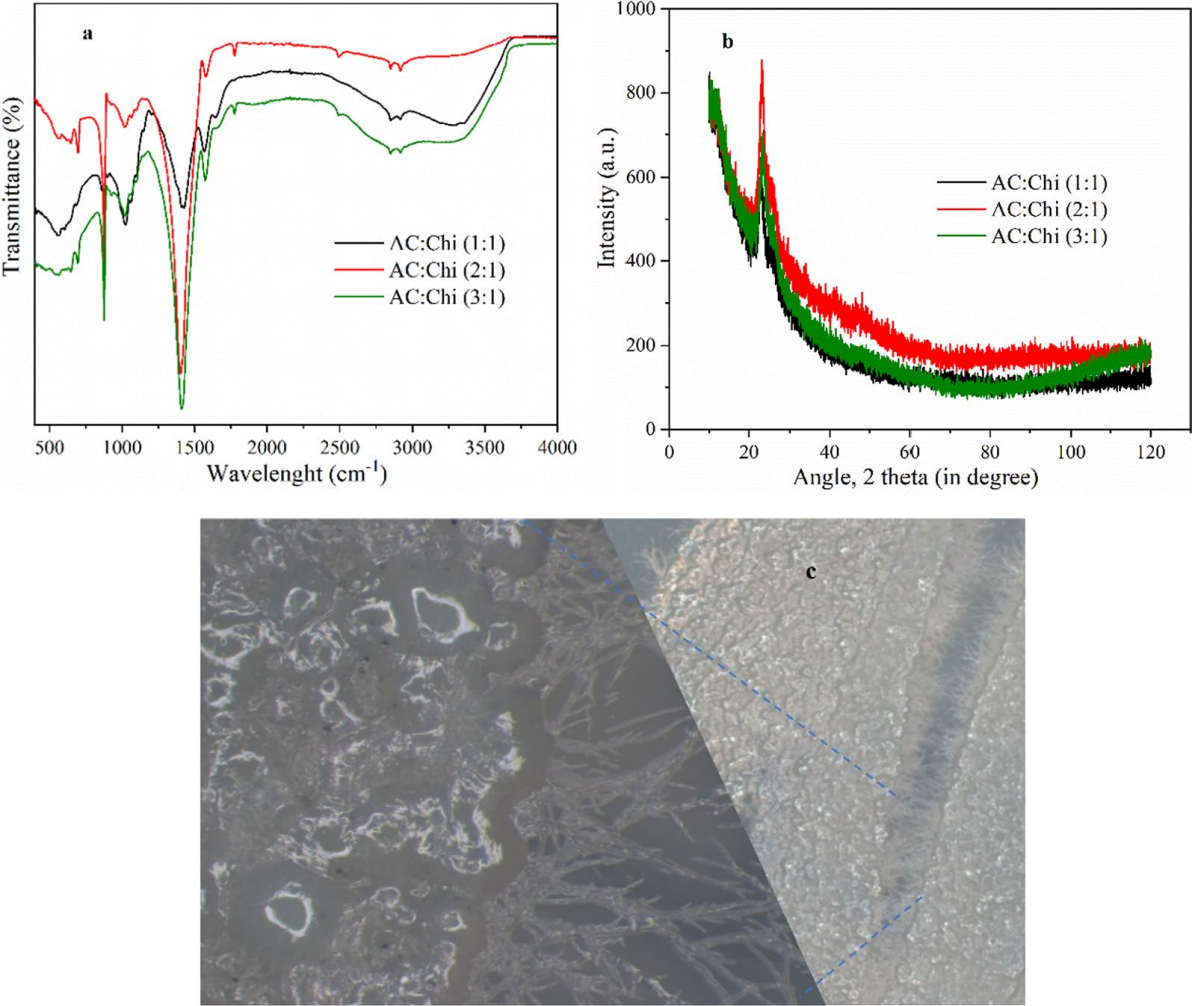
**a** FTIR spectra. **b** XRD analysis spectra for the hybrid beads used as the anode in the microbial fuel cell. **c** Microscopic images of microbial colonies grow on the agar plate

FTIR analysis was used to confirm the surface functional groups on AC:Chi hybrid beads and the spectrum data are shown in Fig. 1a. The primary overlapping area of stretching vibrations of amine and hydroxyl groups shows as a strong broadband in the region 3000–3800 cm^−1^ in AC:Chi hybrid beads [24]. The stretching vibration of the CH_2_ groups is responsible for the smaller peak at 2913 cm^−1^. Furthermore, the amide II band, N–H bending, alcoholic C–O, and C–N stretching may be ascribed to peaks at 1635, 1378, and 1012 cm^−1^, respectively [25]. Moreover, the characteristic peaks observed at 893 cm^−1^ indicates the saccharide structure of chitosan [26]. XRD analysis was also used to characterize the structural properties of AC:Chi hybrid beads, and the pattern is shown in Fig. 1b. The XRD pattern for AC:Chi hybrids portrayed a predominantly strong peak at 22–30°, denoting the amorphous structure of the hybrid materials [27]. The peaks at 45.87 and 56.85 are also found in commercially available chitosan [28]. These results indicate the successful formation of AC:Chi hybrid materials. After inoculation at MFC mode the biofilm formed on the hybrid anodes were taken after the stable voltage is achieved and cultured on the agar plate. The cultured agar plates were studied under a lab-scale microscope and the images are shown in Fig. 1c indicating the presence of microbes growing in colonies.

CHNO elemental analysis was performed to identify the composition of C, H, and N in hybrid beads of activated carbon and chitosan [29]. Table 1 shows the actual and theoretical (in brackets) values of each element in hybrid beads. Based on the results of the elemental analysis, it is possible to infer that hybrid beads were effectively produced in this study.

**Table 1:** Presents the organic elemental analysis of hybrid beads of activated carbon and chitosan

AC:Chi hybrids	Notations	C	H	N
AC:Chi (1:1)	Type I	10.06 ± 0.20 (10.25)	3.45 ± 0.50 (3.95)	1.99 ± 0.10 (2)
AC:Chi (2:1)	Type II	20.98 ± 0.25 (21.50)	4.31 ± 0.80 (5.10)	3.38 ± 0.30 (3.60)
AC:Chi (3:1)	Type III	29.28 ± 0.28 (29.60)	5.81 ± 0.55 (6.40)	3.58 ± 0.44 (3.75)

SEM examination was performed to analyze the morphology of AC:Chi hybrid beads and the findings are shown in the Additional file 1: Fig. SF 2–4). The SEM images of the synthesized hybrid materials showed that the surface was smoother, and the respective biofilm was distributed more uniformly on the surface of each hybrid anode after inoculation. The presence of bacterial colonies can also be seen clearly in the provided images.

### Electrochemical study of hybrid electrodes

**Fig. 2:**
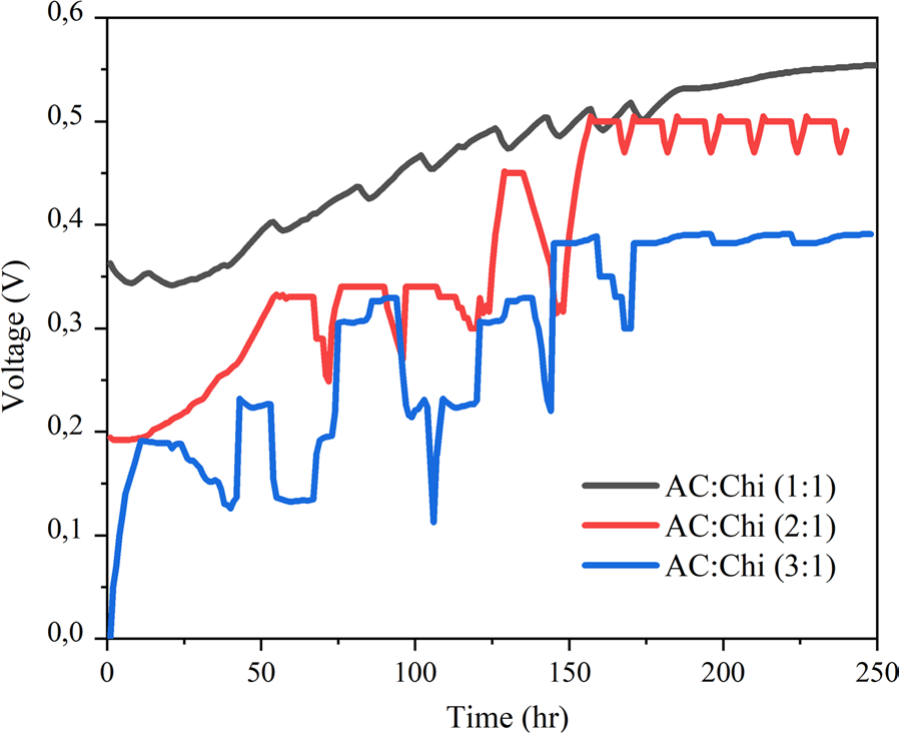
The open cell voltage (OCV) profile vs time (during the inoculation period of 10 days) for activated carbon and chitosan hybrids

The open cell voltage (OCV) recorded during the inoculation period of MFCs with hybrid anodes is shown in Fig. 2. OCV was found to be stable for the AC:Chi (1:1) hybrid anode after 200 h at 0.55 V from the starting point of 0.35 V when feeding with the fresh sludge. On the other hand, the starting voltage of AC:Chi (2:1) hybrid anode was 0.2 V which then increased gradually up to 0.48 V after 170 h. For AC:Chi (3:1), the OCV profile was quite unstable at the beginning starting from 0 V but became stable at 0.35 V after 155 h of inoculation period. When the OCV dropped with the depletion of organics in the treated medium after 78 h, a fresh dosage of the substrate (i.e., acetate) was applied. Following the acetate feeding, there was an immediate increase in OCV. The voltage stayed constant for a few hours before gradually dropping from 0.32 V to 0.25 V and from 0.3 V to 0.2 V in the case of AC:Chi (2:1) and AC: Chi (3:1), respectively. At this point, the enhanced voltage of 0.42 V was observed for AC:Chi (1:1) as compared to the other hybrid anodes. From 180 h of inoculation, the voltage of AC:Chi (1:1) and AC:Chi (3:1) became quite stable as compared to the voltage of AC:Chi (2:1).

**Fig. 3:**
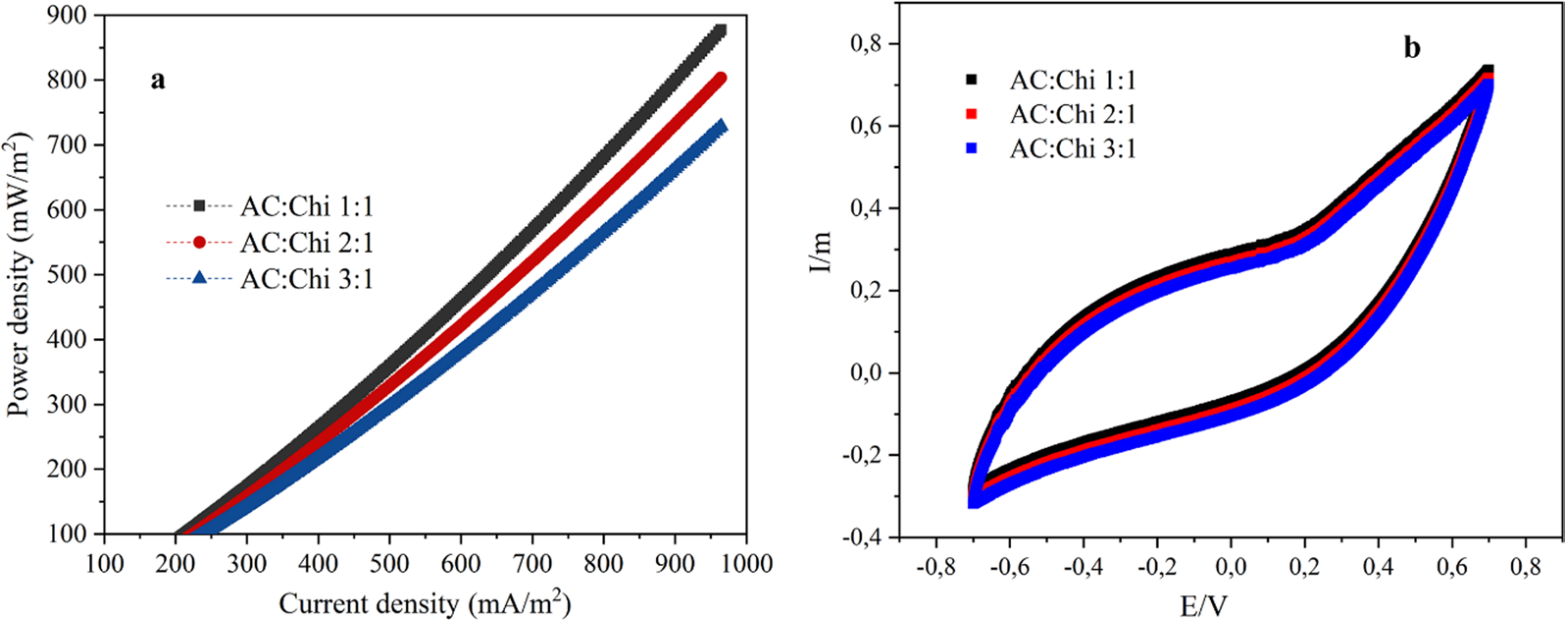
Presents **a** power density vs current density curve for hybrid anodes, **b** cyclic voltammetry plots

The power performance of MFCs with hybrid anodes was compared. The performance of the hybrid anodes was assessed by examining the PCs with a linear sweep CV from − 0.5 to 0.3 V and a 10 mV/s scan. P = V*I, where V and I are the voltage and current generated, was used to compute the power density as shown in Fig. 3a.

The highest power density reached ~ 870 mWm^−2^ for AC:Chi (1:1) (black line) as compared to other hybrid anodes MFCs’. The maximum power density for the AC:Chi (2:1) in Fig. 6 (a) (red line) was ~ 800 mW/m^2^ higher than the maximum power density for the AC:Chi (3:1), for which ~ 700 mW/m^2^ was recorded (see Fig. 3a blue line). The higher power densities for all the hybrid anodes were obtained similarly to the previous study [17]. In addition, cyclic voltammetry (CV) study was carried out to better understand the bioelectrochemical behavior of the systems in various states. The impact of the presence of wastewater and potentiostat settings on the bioelectrochemical behavior of the systems was also investigated. Different forms and patterns of cyclic voltammograms were produced for the three hybrid anodes, as illustrated in Fig. 3b. Because of the varied electrode compositions and conductivities in the presence of wastewater, such discrepancies in the patterns were predicted.

The obtained results from hybrid anodes inoculation suggest that the net energy output was gradually increased as presented in other studies. These results indicate that the hybrid anodes could be used for bioenergy production in MFCs and able to extract elevated energy from the wastewater. This opens a new avenue for resource recovery from wastewater and renewable energy generation.

### COD removal and nutrients recovery from wastewater in MNRC system

**Fig. 4:**
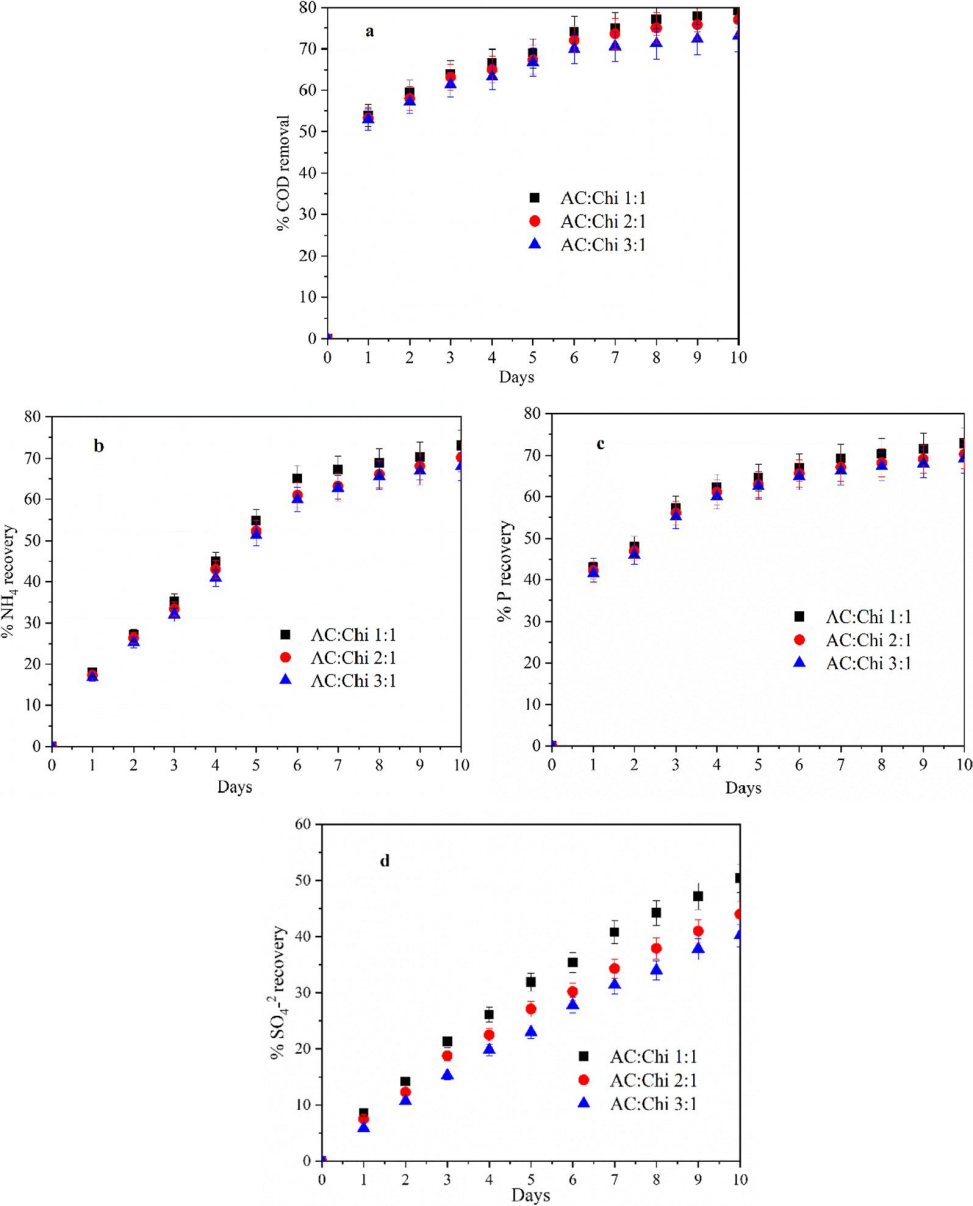
**a** COD removal, **b** % total phosphorus (P) recovery **c** % ammonium recovery, and **d** % SO_4_^2−^ recovery for hybrid anodes in the MNRC system for 10 cycles (i.e., 10 days of operation)

The inoculated electrodes under MFC mode were converted into three-chamber MNRC system as stated above in Sect. 2.5. The conversion of MFC to the MNRC system was adapted from [30], as shown in Fig. 4. To study the COD removal and nutrients recovery from wastewater into recovery solution, MNRC systems were tested with three different hybrid anodes and all the tests were run in duplicates. A 100 mL volume of wastewater comprising TP (2 mg/L), NH_4_^+^ (16 mg/L), and SO_4_^2−^ (25 mg/L) was circulated through anodic and cathodic chambers at a flow rate of 15 mL/min using a peristaltic pump, while a recovery solution comprising 1 g/L of NaCl solution was cycled separately through the recovery chamber. Here, the recovery process was driven by the self-generated electric field between the two electrodes, and no additional external electrical energy was provided for the ions to migrate from the electrode chambers to the recovery chamber and vice-versa. These recovery tests were repeated for 10 days, with each cycle lasting 24 h. This was deemed optimal since a protracted operating cycle might result in ion back diffusion, lowering recovery efficiency. The output voltage was reproducible/repeatable in each operational cycle with the driving force constant throughout the nutrient recovery and concentration tests. It should be noted that in addition to the intensity of the electrical force in the system, the migration process of nutrient ions across the membranes can also be influenced by various factors such as ion concentration (diffusion driven by increased concentration gradient), ion flux (optimized flowrate to enhance the transport of ions), ion size and selectivity of the membrane. The wastewater was recycled for each cycle, but the recovery solution remained constant throughout. The recovery of nutrients from wastewater to the recovery solution (NaCl) and the reduction in COD were continually monitored for 10 days, and the findings are shown in Fig. 4.

The COD removal percent (from wastewater) and recovery percent (nutrients recovery in the recovery solution) were calculated as stated in above Sect. 2.6. The COD content in both chambers was measured during the recovery cycles (Fig. 4a). When employing municipal wastewater, the metabolic activity of the microorganisms presents in the hybrid anodes accommodating MNRC systems consumed approximately ~ 79.2, ~ 77 and ~ 73% of the COD, for AC:Chi (1:1), AC:Chi (2:1), and AC:Chi (3:1), respectively, after 10 days of operation.

As shown in Fig. 4b, the transport of NH_4_^+^ ions from wastewater into the recovery solution was investigated. The recovery percentage of NH_4_^+^ ions rose steadily during 10 days of operation of all the hybrids MNRC systems, reaching maximum of ~ 73% for AC:Chi (1:1) based MNRC systems and ~ 70.2% for AC:Chi (2:1) and ~ 68% for AC:Chi (3:1) MNRC, respectively. The significant increase in NH_4_^+^ ion recovery might potentially be a result of the bacteria decomposing organic materials into ammonia in the anodic chamber [31]. The electron generated during the oxidation of organic materials in the anode chamber was delivered to the cathode for power generation. However, the concentration of NH_4_^+^ in the cathode compartment may be reduced owing to ammonia nitrification into nitrogen [32].

The recovery study of total P was also conducted for developed MNRC system as given in Fig. 4c. In the case of AC:Chi (1:1), ~ 72.9% of P was removed from the wastewater and recovered by the NaCl solution after 10 days of operation. The percentage of recovery of P increased throughout the process for all the hybrid MNRC systems. Similarly, with AC:Chi (2:1) MNRC, ~ 72% of P was removed from the effluent while ~ 70.3% of recovery was achieved. For AC:Chi (3:1) this recovery percentage reaches the value of ~ 69.2 after completion of 10 operation cycles (each cycle lasts for 24 h). Around 2–4 percent of P loss throughout the recovery procedure might be attributed to microbe intake as a nutritional element. The transport of phosphorus from the cathode compartment to the intermediate recovery cell was facilitated via AEM, driven by the internal electric field. Consequently, in the case of hybrid anode materials, the total P concentration in the recovery solution increased with each subsequent operating cycle. Finally, after 10 days (10 operation cycles), the recovery efficiency of hybrid anodes-based MNRC systems climbed highest to roughly ~ 72.9% from starting point for AC: Chi (1:1) MNRC.

Previous research has shown that phosphate precipitation and struvite production occur under alkaline conditions, reducing the performance of membrane and electrode materials [33]. Furthermore, co-removal of sulfur was detected during the recovery process of ammonium and P ions from municipal wastewater. During the 10 days of recovery cycles, however, the highest of ~ 50.4% co-recovery was seen in Fig. 4d for AC:Chi (1:1) MNRC. For other hybrid MNRC systems, this co-recovery of SO_4_^2−^ observed is ~ 44% for AC:Chi (2:1) and ~ 40.2% for AC:Chi (3:1), respectively, after 10 days. According to the literature, sulfur in the form of SO_4_^2−^ ions may be immediately used by the bacterium at the anode [34]. The re-oxidation of sulfur happens on the cathode side, resulting in an increase in sulfur concentration in this chamber and the migration of SO_4_^2−^ ions through AEM from the cathode to the recovery chamber, migrating towards the recovery compartment and concentrating in the recovery solution.

### Microbial community analysis

Mothur MiSeq standard operating procedure (SOP) was followed to determine the microbial diversity over the surface of synthesized electrodes [35]. The diversity of microbial flora over the selected samples taken from the MNRC system (anode brush microbial flora) previously characterized by Mi-sequencing of 16S rRNA gene fragments was determined by Mothur (version 1.41.3) [36]. 16S rRNA gene has several significant properties that make it ideal for the sequencing of all the prokaryotes. Notably, 4 (AC:Chi 1:1), 16 (AC:Chi 2:1) and 12 (AC:Chi 3:1) operational taxonomic units (OTUs) were obtained. The summary of the microbial flora diversity analysis is shown in Table 2.

**Table 2:** DNA sequencing and microbial diversity analysis

Sample	Clean reads	Phylum	Base pairs	Coverage
AC:Chi 1:1	91,130	19	144,788	0.99
AC:Chi 2:1	10,723	17	11,117	0.99
AC:Chi 3:1	14,008	11	14,011	0.98

**Fig. 5:**
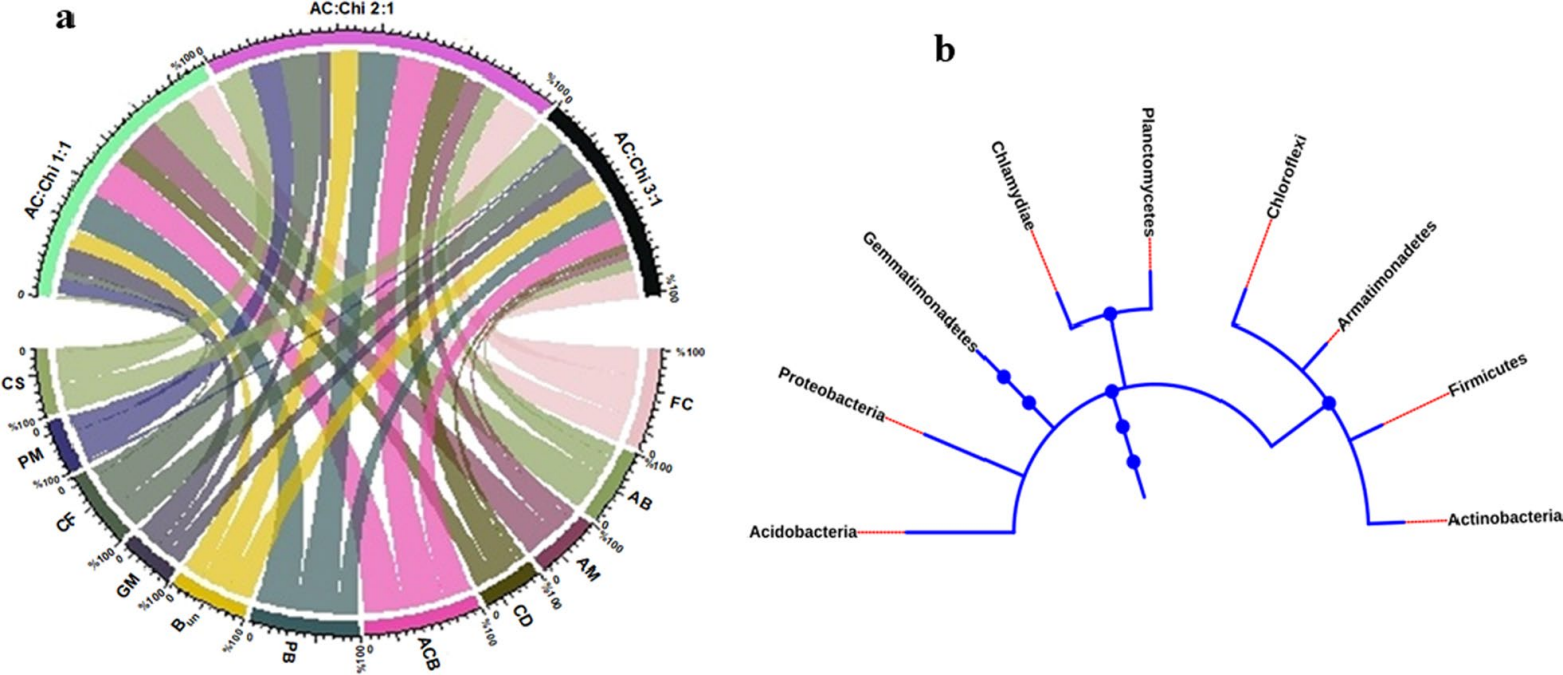
**a** Major phyla over the electrodes-chord diagram using R programming. **b** Phylogenetic tree of the major phyla based on 16S rRNA sequencing

Proteobacteria (PB), firmicutes (FC), Acidobacteria (ACB), Bacteria_unclassified (B_UN_), Actinobacteria (AB), Chloroflexi (CF), Candidatus_Saccharibacteria (CS), Planctomycetes (PM), Gemmatimonadetes (GM), Chlamydiae (CD), and Armatimonadetes (AM) were the dominant phyla over the (AC:Chi 1:1), (AC:Chi 2:1), and (AC:Chi 3:1) anode samples, and the total of the mentioned microbial flora covers 94% of the microbial sequences in the system. The presence of 11 prominent phyla and their distribution in terms of percentage over the anode was represented using a chord diagram (Fig. 5a). The broadness of the band depicts the percentage of the phyla over an anode. Phylogenetic tree (Fig. 5b) represents evolutionary relationships among the Proteobacteria, firmicutes, Acidobacteria, Actinobacteria, Chloroflexi, Planctomycetes, Gemmatimonadetes, Chlamydiae, and Armatimonadetes. The pattern of branching and nodes in a phylogenetic tree reflects a series of evolution.

**Fig. 6:**
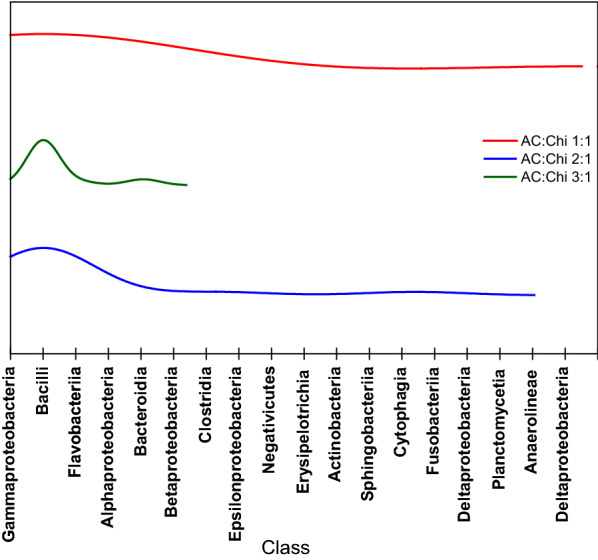
Microbial flora distribution and relative abundance of microbes according to the class over the surface of electrodes

The prepotent microbial population according to the bacterium class were Gammaproteobacteria, Bacilli, Flavobacteriia, Alphaproteobacteria, Bacteroidia, and Betaproteobacteria collectively representing 72% of total bacterial sequences. These classes were the most abundant from the total microbial taxa over the surface of anode samples. The density of microbial class over the (AC:Chi 1:1), (AC:Chi 2:1), and (AC:Chi 3:1) anode samples is shown in Fig. 6. It was observed that (AC:Chi 1:1) and (AC:Chi 2:1) have diverse microbial flora but in the case of (AC:Chi 3:1) anode Gammaproteobacteria, Bacilli and Flavobacteriia were present in higher density. In our tested MNRC systems, the feed and substrate conditions were all maintained the same except for the developed hybrid anodes. Hence, it can be said from Table 2 and Fig. 6 that the increase in chitosan content in the electrode matrix improved biofilm formation and microbial diversity which in turn positively influenced the voltage generation of the respective systems, observed similarly in other works [37, 38]. The diverse and enhanced microbial consortia present on the surface of the hybrid AC:Chi 1:1 and 2:1 anodes produced a constant voltage of 0.55 V and 0.48 V, respectively, in comparison to 0.35 V of hybrid AC:Chi (3:1) with the lesser diverse microbial consortia.

### Economic value

Simple setup and the use of low-cost components are critical for the effective deployment of a microbial nutrient recovery system in the actual world. The goal of this research is to create a simple and easy-to-manage microbial nutrient recovery system with sustainable bioenergy generation, effective nutrient recovery from a given source, and clean water generation for subsequent reuse. The further development in the above-described studies, e.g., synthesis of hybrid beads of activated carbon and chitosan with different ratios will further make advancement in the present output voltage and nutrient recovery process concerning time. Also, additional tests to obtain higher nutrient concentrates should be performed by increasing the current in the system by external means. Future works should be designed to improve the performance of this system by testing higher COD containing wastewater, enlarged reactor, improved design of the chambers to reduce the solution resistance, and superior ion-selective membrane for enhanced nutrient recovery.

## Conclusion

This work was the first to combine the activated carbon and chitosan hybrid anodes in the MFC and MNRC system, which eliminated most of the organic contaminants (COD), nutrients including ammonium, P, and sulfate from the municipal wastewater, with microbial culture for deeper and more sustainable nutrient recovery. The presence of hybrid anodes with different ratios in the MFC process was shown to be efficient in terms of energy production and high power densities when compared to the previously reported studies. Our findings contributed to a better understanding of the foundations of the MFC, allowing it to be tailored for specific purposes and applications. In addition to nutrition recovery, the hybrid anode accommodating systems provided constant voltage outputs of 0.55 V and 0.48 V, and 0.35 V for AC:Chi (1:1), AC:Chi (2:1), and AC:Chi (3:1), respectively. The inoculated hybrid anode displayed efficient COD removal (73–79%) as well as simultaneous recovery of nutrients in the form of NH_4_^+^ (~ 73%) and P (~ 72.9%) ions from municipal wastewater. Furthermore, the co-removal of interfering ions like SO_4_^2−^ ions was found to be very low (~ 50%) in contrast to target nutrients like P and NH_4_^+^ ions. Overall, the consistent voltage response and higher power density values observed with the hybrid anode-based MFC-MNRC system are promising and warrant further improvement in future investigations. In the future study, the effects of the wet-proofed AC cathode, catalyst and/or membrane selection, and cell architecture on system power output should be researched and improved for the proposed hybrid anode MNRC.

## Materials and methods

### Preparation of hybrids of activated carbon and chitosan as anode material

The hybrid gel beads of activated carbon (AC) (Charcoal activated Norit^®^, CAS No.: 7440-44-0; diameter: ~ 3 mm) and chitosan (Chi) (medium molecular weight; CAS No.: 9012-76-4; 200–800 cP viscosity; 75–85% deacetylated; Sigma Aldrich) was prepared using prior described techniques in literature studies with some modifications. The structure of hybrid beads is given in the Additional file 1: Fig. SF1.

**Fig. 7:**
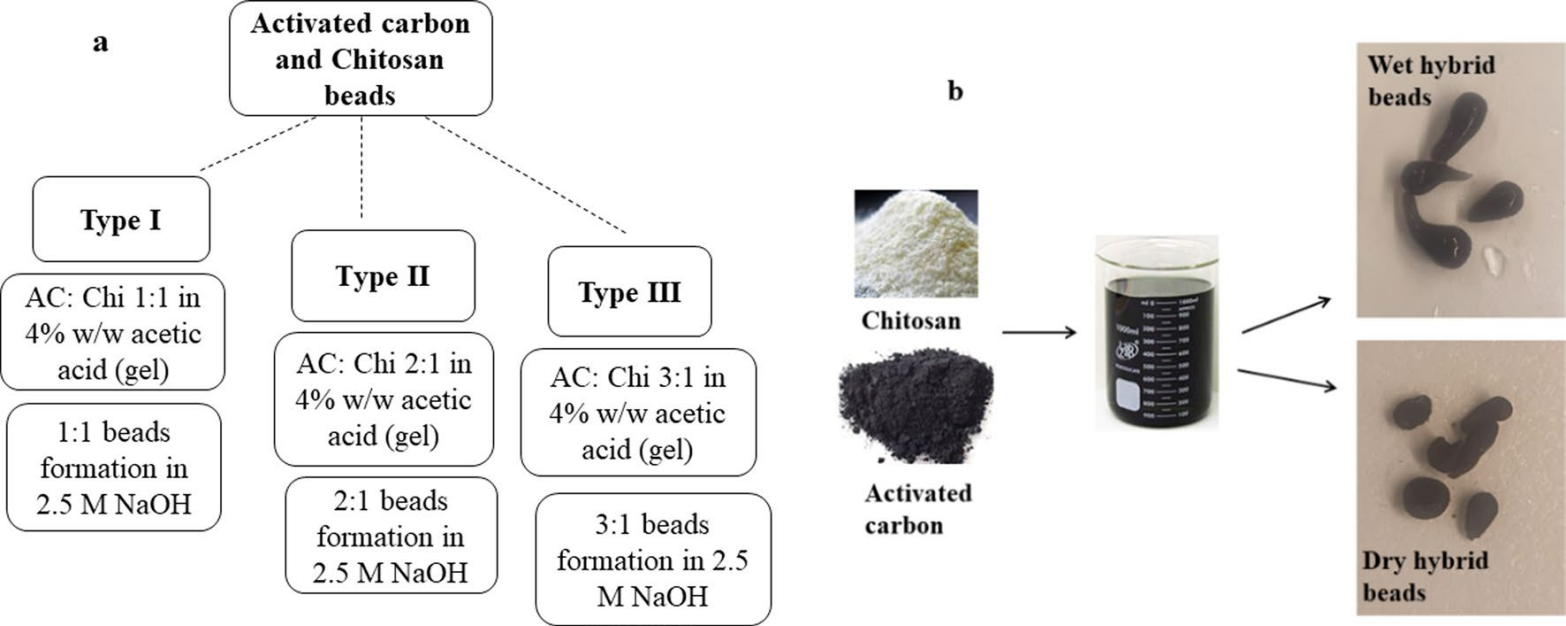
**a** Formation of three types of hybrid beads of activated carbon and chitosan used in the present study. **b** Wet hybrid beads after formation and dry hybrid beads after dried in the oven

In this study, three types of hybrid beads were prepared to depend upon the concentration of AC (pellets were then thoroughly rinsed with deionized water) mixed with chitosan. The beads were described as Type I, II, and III in this article as shown in Fig. 7a. For Type I beads equal volume of both AC and Chi were mixed into 4% w/w of glacial acetic acid (Sigma Aldrich, Cas No.: 64-19-7; MW.: 60.05) and the resulting viscous gel was dropped into a 2.5 M sodium hydroxide solution bath. The form viscous beads were left undisturbed into NaOH bath for a couple of hours. This process is followed by several times washing of beads with water for removal of the excessive basic solution. Following this procedure other two types of hybrid beads such as Type II (AC 2:Chi 1) and Type III (AC 3:Chi 1) were also prepared, to acquire sound knowledge on the effect of their ability to accommodate microbes and recovery efficiency of nutrients from given electrolyte medium. In the present study prepared hybrid beads of AC and Chi were used as anode material as shown in Fig. 7b. Commercially available granular AC and medium molecular weight chitosan powder were used for this purpose.

### Preparation of air cathode electrode

**Fig. 8:**
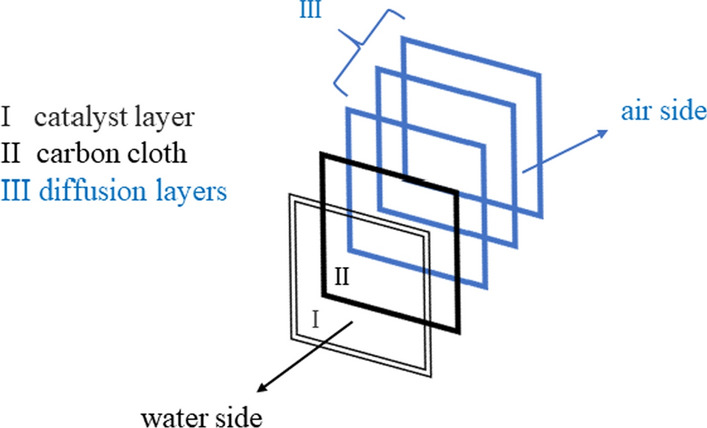
Assembly of the cathode used in the present study: catalyst layer (towards the water-facing side) and DLs (towards the air-facing side) in MFC

The detailed preparation of the cathode electrode was given in the previous study [30]**.** For the preparation of cathode Carbon Cloth, Wet Proofed (Fuel Cell Store, product code: 7302008) was used as a projected surface area of 7 cm^2^ as shown in Fig. 8. A carbon base layer was spread evenly to the airside of the carbon cloth by combining carbon black (Vulcan XC 72: Fuel Cell Earth: SKU: CV-XC72-50) with 30 wt. percent Polytetrafluoroethylene (PTFE; 60 wt. percent dispersed in water) (Sigma Aldrich, CAS No.: 9002-84-0). The applied carbon base-polytetrafluoroethylene (PTFE) coating over carbon cloth was air-dried for 2 h before being heated in a furnace (Nabertherm B 180) at 370 °C for 30 min. Additional diffusion layers (DLs) were loaded onto a carbon base layer after cooling at room temperature by brushing the PTFE solution uniformly. After drying the layers at ambient temperature for 10–15 min, they were heated in a furnace (Nabertherm B 180) at 370 °C for 10 min [2]. In this investigation, four DLs of PTFE were applied. The platinum (Pt) catalyst layer was created on the opposite side, i.e., the water-facing side of the cathode, by combining 10% Pt/carbon (10 wt. percent Pt/C) (Fuel Cell Store, product code.: 591078) with Nafion (Sigma Aldrich, CAS No.: 31175-20-9) functioning as a binder [39]. The carbon fabric was then dried for 24 h before being used in the reactor.

### MFC reactors construction

The cathode is on the right side of the single-cell MFC reactor, with the hybrid anodes occupying the remaining area. The cathode employed in this study has a specific surface area of 7 cm^2^. External connections were made using titanium (Ti) wire [(annealed, 0.5 mm), (CAS No.: 7440-32-6) VWR)] weaved into the carbon cloth. The external resistance of 1000 Ω was applied.

### Hybrid anodes inoculation at MFC configuration

All the hybrid anodes in this investigation were inoculated in a single-chamber MFC configuration before being placed in the MNRC system. The MFC reactors used in the present study for inoculation have a volume of 28 mL. They were non-conductive made up of plexiglass material. For inoculation, real activated sludge for primary aeration tank (effluents) from the local wastewater treatment plant located in Mikkeli, (Kenkävero, Finland) was used. A sampling of wastewater was conducted from the same location, but at different periods and used to inoculate all batches of hybrid anodes accommodating MFC that were initiated at different points. At the start-up point of inoculation, the hybrid anode material already placed in the MFC reactor was inoculated using only fresh wastewater for a whole operation cycle (that is 24 h). After that for the next two operation cycles, similar reactors were fed with a mixture of wastewater and synthetic media (1:1). The composition of synthetic media includes sodium acetate (1 g/L) that is dissolved in 50 mM of phosphate buffer (Na_2_HPO_4_, 4.58 g/L; NaH_2_PHO_4_-H_2_O 2.45 g/ L; NH_4_Cl 0.31 g/L; KCl 0.13 g/L; trace minerals and metals solution and vitamins; conductivity of 6.95 mS/cm). Later, only the acetate medium (1 g/L) was added. The same procedure of inoculation was repeated with all the anodes until an optimum voltage value was achieved. The wastewater utilized for the initial inoculation had a chemical oxygen demand (COD) of 718 mg/L, a pH range of 7.01—7.2 with a conductivity of 8.66 mS/cm. All the reactors were run in parallel in fed-batch mode at a room temperature of 22 ± 2 °C.

### Conversion of MFC into MNRC system

**Fig. 9:**
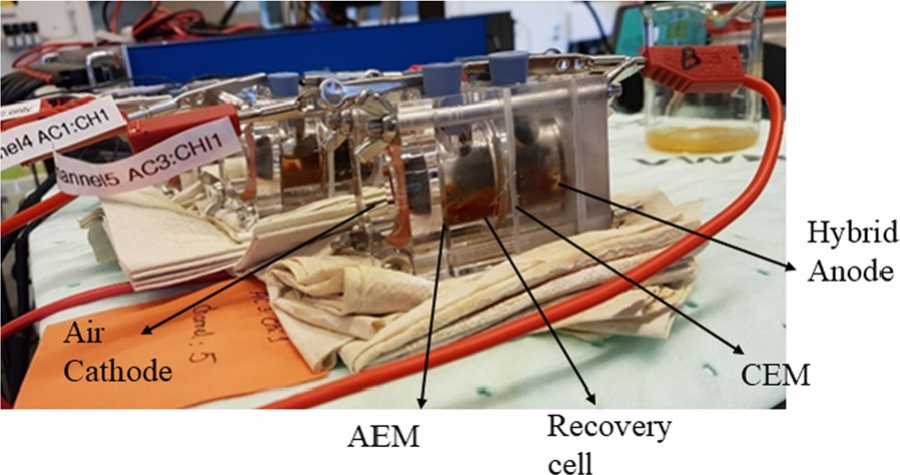
MNRC system with hybrid anode at right and air cathode at left used in the present study

The single-chamber MFC reactor is converted into a three-chamber MNRC system consisting of an anode chamber (on left) containing inoculated hybrid anodes (Type I, II, and III) as shown in above Fig. 7b and a cathode chamber (on right) accommodating an air–cathode (Fig. 8) and a middle recovery chamber of the same volume (28 mL) as both anode and cathode chamber was placed in between. As shown in Fig. 9, an anion exchange membrane (AEM, AMI-7001S, Membrane International Inc.) was placed between the cathode and the recovery chamber, and a cation exchange membrane with specification (CEM, CMI7000S, Membrane International Inc.) was placed adjacent to the anode and the recovery chamber. To prevent internal and exterior leakage, silicon rings were positioned between the membranes. To facilitate the data recovery during the nutrients and COD removal process the electrodes were connected to the titanium wire.

### Characterization study

To understand the structure and morphology of AC and Chi hybrids, Fourier Transform Infrared Spectroscopy (FTIR, Bruker Vertex 70, 400 to 4000 cm^−1^ using 4 cm^−1^ resolution and a rate of 100 scans per sample) was used to investigate the surface functional groups of hybrid anodes. To estimate the contents of % Carbon, Hydrogen, and Nitrogen, CHNSO Organic Elemental Analyzer (Flash 2000, Thermo Scientific) was employed. Each sample was weighed into a tin capsule at a rate of 2.0 mg. The tin capsule was then placed in a combustion reactor for analysis. The XRD studies used in the present study were performed by X-ray diffraction spectroscopy (XRD, PANalytical instrument with Empyrean program using CoKα irradiation at λ = 1.78 Å, 40 mV and 30 mA). The microstructure of hybrid anodes was characterized by scanning electron microscopy (SEM) (Hitachi. SU3500, Japan) using different acceleration voltages of 0.1–0.7 V. The biofilm produced at the surface of hybrids anodes was taken from it and cultured on an agar plate and the cultured microbial colonies were also checked using a lab-scale microscope.

Data acquisition logger (Pico datalogger ADC-20) monitored the voltage (V) profiles of all the MFC reactors throughout the inoculation time. When the voltage of fuel cells remained stable the power density was calculated by changing the external resistor from an open-cell circuit to 1000 ohms. The electrodes were electrochemically characterized utilizing cyclic voltammetry (CV) analysis with a Potentiostat (IVIUM OctoStat5000) at a scan rate of 10 mVs^−1^ in the MFC reactors [40]. The COD of wastewater was determined using the Hach cuvette test (0–1000 mg/L COD) before and after each operating cycle. Shimadzu Ion chromatography for anions and cations was used to quantify the removal efficiency of nutrients from wastewater after each operating cycle (one cycle lasts 24 h). After each operation cycle, the performance efficiency of hybrid anode MNRC was assessed based on COD removal efficiency from wastewater, power density generation, and recovery efficiency of nutrients from wastewater to the recovery solution as given in the Additional file 1: Eqs (A.1) and (A.2).

### Microbial study

After the completion of the inoculation period, biofilm was removed from the hybrid anode electrodes and cultivated for DNA isolation. To amplify the microbial DNA for the 16S rRNA amplicon Mi-sequencing, Polymerase chain reaction (PCR) was used. In the current investigation, 16S rRNA amplicon Mi-sequencing was used to detect the microbial populations. For Mi-sequencing, the normal operating protocol was followed. Each sample's DNA was amplified using a set of primers (ACTCC-TACGGGAGGCAGCAG and GGA TACHVGGGTWTCTAAT) that targeted the 16S rRNA gene.

## Supplementary Information

**Additional file 1.** Chemical structure of hybrid anodes, Calculation of the COD removal and nutrients recovery from wastewater and, Results from the SEM analysis of AC:Chi (1:1), (AC:Chi (2:1) and AC:Chi (3:1) hybrids anodes.

## Data Availability

The data that support the findings of this study are available upon request from the corresponding author.

## Abbreviations

AC: Activated carbon
Chi: Chitosan
AC:Chi: Activated carbon and chitosan hybrids
AEM: Anion exchange membrane
CEM: Cation exchange membrane
conc.: Concentration
COD: Chemical oxygen demand
DLs: Diffusion layers
FTIR: Fourier transform infrared spectroscopy
IC: Ion chromatography
*I*: Current
MFC: Microbial fuel cell
MNRC: Microbial nutrient recovery cell
mV: Millivolts
mW/m^2^: Milliwatts per square meter
NaAc: Sodium acetate
NH_4_^+^: Ammonium
OCV: Open cell voltage
P: Phosphorus
PBS: Phosphate buffer
Pt: Platinum
PTEF: Polytetrafluoroethylene
RS: Recovery solution
SEM: Scanning electron microscopy
SO_4_^2^^−^: Sulfate
*V*: Voltage
XRD: X-ray diffraction spectroscopy
