# Supplementary material for: Enhanced bioenergy and nutrients recovery from wastewater using hybrid anodes in microbial nutrient recovery system
Source: Biotechnol Biofuels Bioprod. 2022 Feb 17;15:19. doi: 10.1186/s13068-022-02116-y (PMC8855553; doi:10.1186/s13068-022-02116-y)
Supplement: Supplementary file 1 — Additional file 1. Chemical structure of hybrid anodes, Calculation of the COD removal and nutrients recovery from wastewater and, Results from the SEM analysis of AC:Chi (1:1), (AC:Chi (2:1) and AC:Chi (3:1) hybrids anodes. [file 13068_2022_2116_MOESM1_ESM.docx]

**Enhanced bioenergy and nutrients recovery from wastewater using hybrid anodes in microbial nutrient recovery system**

Kanwal Shahid^a^*, Deepika Lakshmi Ramasamy^b^, Parminder Kaur^c^, Mika Sillanpää^d,e,f,g,h^, Arto Pihlajamäki^a^

^a^Department of Separation Science, School of Engineering Science, Lappeenranta-Lahti University of Technology, Sammonkatu 12, FI-50130 Mikkeli, Finland

^b^Department 4MAT, Université libre de Bruxelles, Avenue F.D. Roosevelt, 50. CP 165/63 1050 Bruxelles, Belgium

^c^Department of Chemical and Metallurgical Engineering, Aalto University, Aalto, Finland.

^d^Chemistry Department, College of Science, King Saud University, Riyadh 11451, Saudi Arabia

^e^School of Resources and Environment, University of Electronic Science and Technology of China (UESTC), NO. 2006, Xiyuan Ave., West High-Tech Zone, Chengdu, Sichuan 611731, P.R. China

^f^Faculty of Science and Technology, School of Applied Physics, University Kebangsaan Malaysia, 43600, Bangi, Selangor, Malaysia

^g^School of Chemistry, Shoolini University, Solan, Himachal Pradesh, 173229, India

^h^Department of Biological and Chemical Engineering, Aarhus University, Nørrebrogade 44, 8000 Aarhus C, Denmark

*Corresponding authors: [*kanwal.shahid@lut.fi*](mailto:kanwal.shahid@lut.fi)





Fig. SF1. Structure of activated carbon and chitosan hybrid beads

$\% {Re}_{(Nu)}=\frac{{RS}_{conc. (fi)}-{RS}_{conc. (ini)}}{{RS}_{conc.(fi)}}*100$ Eq. (A1)

$\% {COD}_{(Rem)}=\frac{C_{o (WW)}-C_{f(WW)}}{C_{o(WW)}}*100$ Eq. (A2)

Eq. (A.1), *Re_(Nu)_* denotes recovery efficiency of nutrients (phosphate, and ammonium, and sulphate) from wastewater to recovery solution, and in Eq (A.2) *COD_(Rem)_* indicates COD removal efficiency from wastewater after completion of each operating cycle. whereas the *C_O (WW)_* and *C_f (WW)_* are initial and final concentrations of targeted ions in the wastewater, respectively. ${RS}_{conc. (fi)}$ and ${RS}_{conc. (ini)}$ presents the final concentration of a targeted ion in recovery solution and initial concentration of targeted ion in recovery solution, respectively.










Fig. SF2. Electron microscope images of biofilm sample taken from AC:Chi (1:1)










Fig. SF3. Electron microscope images of biofilm sample taken from AC:Chi (2:1)










Fig. SF4. Electron microscope images of biofilm sample taken from AC:Chi (3:1)
